# Mirizzi Syndrome Mimicking Cholangiocarcinoma Complicated by Infected Biloma Formation Following Complex Biliary Intervention: A Case Report

**DOI:** 10.7759/cureus.109763

**Published:** 2026-05-27

**Authors:** Wilfredo J Javier-Rojas, Vamsee Mupparaju, Douglas M Rivera-Agosto, Yizhi Lin

**Affiliations:** 1 Department of Internal Medicine, Morsani College of Medicine Graduate Medical Education (GME), HCA Florida Blake Hospital, HCA Healthcare/University of South Florida (USF), Bradenton, USA; 2 Department of Gastroenterology, Morsani College of Medicine Graduate Medical Education (GME), HCA Florida Citrus Hospital, HCA Healthcare/University of South Florida (USF), Inverness, USA

**Keywords:** biliary obstruction, biliary sepsis, biliary stent removal, biloma, endoscopic retrograde cholangiopancreatography (ercp), gallstone disease (gsd), indocyanine green cholangiography, minimally invasive surgery, mirizzi's syndrome, subtotal fenestrating cholecystectomy

## Abstract

Mirizzi syndrome is a rare but clinically significant complication of chronic cholelithiasis. It is characterized by extrinsic compression of the common hepatic duct (CHD) by an impacted gallstone in the gallbladder neck or cystic duct. Although rare, this condition can closely mimic cholangiocarcinoma, particularly in the presence of elevated tumor markers or atypical imaging findings. Timely recognition is essential, as misdiagnosis may lead to inappropriate surgical or oncologic interventions.

We present a diagnostically challenging case of Type I Mirizzi syndrome complicated by infected biloma formation occurring shortly after elective biliary stent removal in the setting of prior subtotal cholecystectomy and a retained common bile duct (CBD) stone. A 53-year-old female presented with nausea, vomiting, weight loss, and elevated transaminases. Imaging revealed biliary ductal dilatation and abnormal gallbladder morphology, raising suspicion for a biliary tract malignancy. CA 19-9 was mildly elevated initially but decreased following biliary decompression, further supporting a benign obstructive process rather than malignancy. Magnetic resonance imaging (MRI) with magnetic resonance cholangiopancreatography (MRCP) protocol demonstrated distal CBD compression by an enlarged cystic duct containing a filling defect, consistent with Type I Mirizzi syndrome. The diagnosis was confirmed via endoscopic retrograde cholangiopancreatography (ERCP), which also served as the initial therapeutic intervention with biliary sphincterotomy, stone extraction, and stent placement. The patient subsequently underwent robotic-assisted cholecystectomy with a subtotal fenestrating technique due to difficult anatomy, with an initially uncomplicated postoperative course.

Seven weeks after cholecystectomy, the patient underwent elective biliary stent removal and re-presented within 48 hours with abdominal pain and signs of biliary sepsis. Imaging confirmed a complex fluid collection in the gallbladder fossa consistent with a biloma, along with recurrent CBD dilation and a retained CBD stone contributing to recurrent biliary obstruction. The patient underwent percutaneous drainage followed by repeat ERCP with stone extraction. Cultures of biloma fluid and blood grew *Klebsiella pneumoniae* and *Escherichia coli*, respectively, consistent with biliary sepsis. With antibiotic therapy and successful drainage, the patient improved clinically and was discharged with outpatient follow-up.

This case highlights the diagnostic complexity of Mirizzi syndrome, particularly its ability to masquerade as malignancy due to overlapping radiologic and biochemical features. It also underscores the importance of recognizing infected biloma formation and recurrent biliary obstruction as potential complications following complex biliary interventions, particularly in patients undergoing subtotal cholecystectomy and biliary stent management. Management is inherently multidisciplinary, involving interventional radiology, gastroenterology, and surgery. Early recognition and timely intervention are essential to prevent infectious sequelae and optimize outcomes.

## Introduction

Mirizzi syndrome is a rare complication of chronic cholelithiasis, reported in approximately 0.05% to 4% of patients undergoing surgery for gallstone disease, resulting from impaction of a gallstone within the cystic duct, gallbladder neck, or infundibulum (Hartmann’s pouch), leading to extrinsic compression of the common hepatic duct (CHD) and subsequent biliary obstruction [1]. Persistent obstruction may result in upstream biliary dilation and progressive inflammatory changes, which can further complicate the clinical course. Mirizzi syndrome is commonly classified using the Csendes classification system, which stratifies disease into Types I through V (Va and Vb), based on the degree of bile duct involvement and the presence or absence of cholecystocholedochal and cholecystoenteric fistulas [1].

Clinically and radiographically, Mirizzi syndrome may closely mimic biliary tract malignancies, particularly cholangiocarcinoma, posing a significant diagnostic challenge [2-4]. Several published reports have described Mirizzi syndrome masquerading as biliary tract malignancy, underscoring the importance of careful interpretation of imaging studies and clinical findings. Cross-sectional and cholangiographic imaging play a pivotal role in the diagnostic workup.

Definitive management of Mirizzi syndrome typically necessitates invasive intervention, most commonly surgical, though endoscopic retrograde cholangiopancreatography (ERCP) is often employed diagnostically and therapeutically. These interventions, however, carry the risk of uncommon yet serious complications, including bilomas, defined as localized collections of bile outside the biliary tree. Bilomas may result from inadvertent bile leakage during procedures such as cholecystectomy or ERCP. Although biloma formation is a recognized postoperative complication of hepatobiliary interventions, clinically significant infected bilomas remain relatively uncommon and may present important diagnostic and therapeutic challenges, particularly in the setting of complex biliary disease and postoperative bile leaks [5]. Although well described in the postoperative setting, their occurrence in the context of Mirizzi syndrome remains uncommon and may further complicate management. Prompt recognition and appropriate management are critical to mitigate infectious complications and optimize clinical outcomes. 

This case is notable for the combination of Mirizzi syndrome initially concerning for malignancy, complex subtotal fenestrating cholecystectomy, retained common bile duct (CBD) stone, and subsequent infected biloma formation following biliary intervention, highlighting the diagnostic and postoperative challenges associated with complex biliary disease.

## Case presentation

A 53-year-old female with a past medical history of type 2 diabetes mellitus and essential hypertension presented with a four-day history of persistent nausea, non-bilious vomiting, decreased oral intake, and low-grade intermittent fevers. She also reported epigastric discomfort described as a dull, aching pain worsened by meals, associated with postprandial fullness. Additionally, she endorsed an unintentional 15-pound weight loss over the preceding two months and generalized fatigue. Her body mass index (BMI) was 17.3 kg/m². She denied jaundice, changes in stool or urine color, melena, hematemesis, or recent travel. She denied any prior history of symptomatic gallstone disease, biliary colic, pancreatitis, cholangitis, or previous hepatobiliary interventions. 

On presentation, the patient appeared mildly ill but was afebrile (98.4°F), with a heart rate of 86 beats per minute, respiratory rate of 17 breaths per minute, blood pressure of 143/84 mmHg, and oxygen saturation of 98% on room air. Physical examination was notable for dry oral mucosa and mild epigastric tenderness without rebound or guarding. Murphy’s sign was negative. There was no hepatomegaly, palpable masses, peripheral edema, or lymphadenopathy.

Initial laboratory evaluation demonstrated mild hyponatremia (sodium 135 mmol/L, reference range: 136-145 mmol/L) and a mixed hepatocellular-cholestatic pattern of liver injury, with markedly elevated aspartate aminotransferase (AST 1792 U/L, 15-37 U/L) and alanine aminotransferase (ALT 1098 U/L, 12-56 U/L), alongside elevated alkaline phosphatase (ALP 767 U/L, 45-117 U/L) and gamma-glutamyl transpeptidase (GGT 922 U/L, 5-55 U/L). Total bilirubin was mildly elevated at 1.9 mg/dL (0.2-1.0 mg/dL). The degree of transaminitis was disproportionate to the level of cholestasis and was suggestive of acute biliary obstruction, a pattern that may occur in transient choledocholithiasis or Mirizzi syndrome. The white blood cell (WBC) count was 5.8 × 10³/µL (4.0-10.5 × 10³/µL) with neutrophilic predominance. Table 1 provides a detailed summary of the comprehensive metabolic panel (CMP) and complete blood count (CBC) with differential. Lipase, lactic acid, and viral hepatitis serologies were within normal limits (Table 2).

**Table 1:** Comprehensive Metabolic Panel and Complete Blood Count with Differential

Test Category	Test	Value	Units	Reference Range
Complete Blood Count (CBC)	White Cell Count (WBC)	5.8	×10³/µL	4.0 – 10.5
	Hemoglobin	14.6	g/dL	11.2 – 15.7
	Platelets	366	×10³/µL	150 – 450
CBC with Differential	Immature Granulocytes	0.2	%	0.0 – 0.4
	Neutrophils	74.8	%	34.0 – 71.1
	Lymphocytes	17.2	%	19.3 – 51.7
	Monocytes	6.5	%	4.7 – 12.5
	Eosinophils	0.3	%	0.7 – 5.8
	Basophils	1.0	%	0.1 – 1.2
Comprehensive Metabolic Panel (CMP)	Sodium	135	mmol/L	136 – 145
	Potassium	3.5	mmol/L	3.5 – 5.1
	Chloride	97	mmol/L	98 – 107
	Carbon Dioxide	25.5	mmol/L	21.0 – 32.0
	Blood Urea Nitrogen (BUN)	9	mg/dL	7 – 18
	Creatinine	0.6	mg/dL	0.6 – 1.0
	Glucose	185	mg/dL	74 – 106
	Total Bilirubin	1.9	mg/dL	0.2 – 1.0
	Aspartate Aminotransferase (AST)	1792	U/L	15 – 37
	Alanine Aminotransferase (ALT)	1098	U/L	12 – 56
	Alkaline Phosphatase (ALP)	767	U/L	45 – 117
	Gamma Glutamyl Transpeptidase (GGT)	922	U/L	5 – 55
	Albumin	4.5	g/dL	3.4 – 5.0

**Table 2:** Additional Laboratory Evaluation: Lipase, Lactic Acid, and Viral Hepatitis Serologies

Test Category	Test	Value	Units	Reference Range / Result
Pancreatic Enzymes	Lipase	79	U/L	73 – 393
Metabolic Marker	Lactic Acid Level	1.0	mmol/L	0.4 – 2.0
Hepatitis Panel	Hepatitis A IgM Antibody	Negative	—	Negative
	Hepatitis B Surface Antigen	Negative	—	Negative
	Hepatitis B Core IgM Antibody	Negative	—	Negative
	Hepatitis C Antibody	Negative	—	Negative

Given concern for a possible infectious intra-abdominal process, empiric intravenous piperacillin-tazobactam was initiated. A contrast-enhanced computed tomography (CT) scan of the abdomen and pelvis (Figure 1) demonstrated mild intrahepatic biliary ductal dilatation and an abnormal gallbladder with irregular wall thickening and enhancement, raising suspicion for a neoplastic process. The cystic duct measured 7.6 mm and was noted to arise from a relatively low insertion. The hepatic duct measured 10 mm. The distal CBD was of normal caliber.

**Figure 1:**
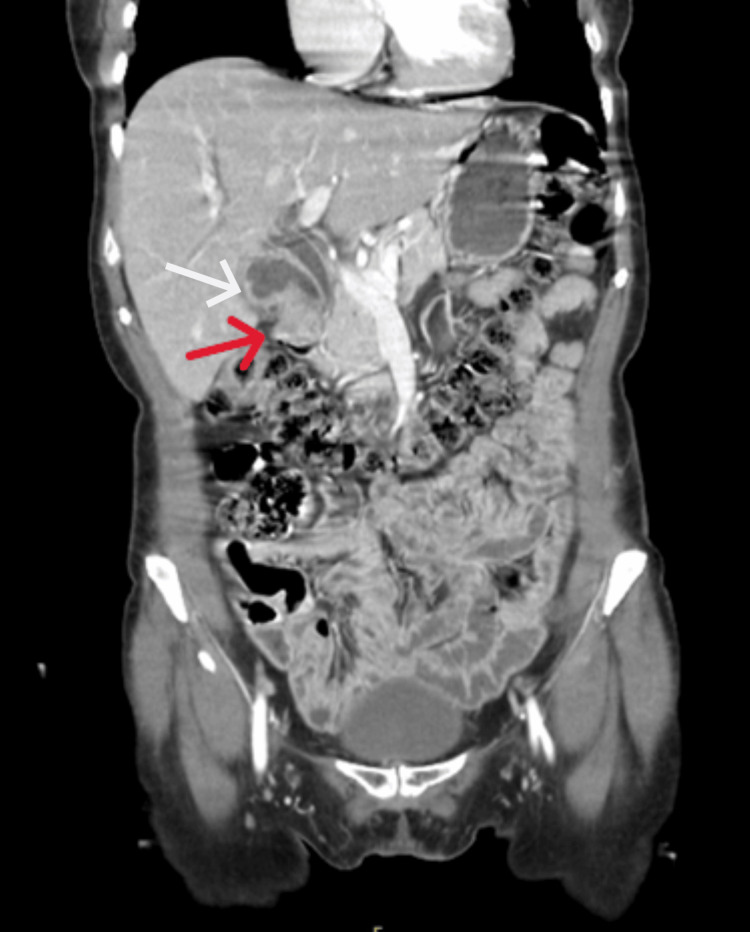
Contrast-enhanced computed tomography (CT) scan of the abdomen and pelvis Contrast-enhanced CT of the abdomen and pelvis demonstrating mild intrahepatic biliary ductal dilatation and irregular gallbladder wall thickening (white arrow) with associated enhancement. The cystic duct appears distended (7.6 mm) with a low insertion. The common hepatic duct measures 10 mm, while the distal common bile duct is of normal caliber. Overall findings may raise concern for a neoplastic process (red arrow).

Given concern for malignancy, a staging chest CT was performed and showed no suspicious pulmonary lesions, pleural effusion, or pleural thickening. Erythrocyte sedimentation rate (ESR) was elevated at 32 mm/hr, whereas C-reactive protein (CRP) remained within normal range. Tumor markers revealed a mildly elevated CA 19-9 of 114 U/mL; carcinoembryonic antigen (CEA) and alpha-fetoprotein (AFP) were within normal limits at 2.8 ng/mL and 3.8 ng/mL, respectively (Table 3). Hematology/Oncology and General Surgery were consulted to evaluate for possible cholangiocarcinoma or gallbladder carcinoma.

**Table 3:** Tumor and Inflammatory Marker Evaluation

Test Category	Test	Value	Units	Reference Range
Tumor Markers	Alpha Fetoprotein (AFP)	3.8	ng/mL	0.0 – 8.0
	Carcinoembryonic Antigen (CEA)	2.8	ng/mL	0 – 5.0
	CA 19-9 (Carbohydrate Antigen 19-9)	114.0	U/mL	0.0 – 30.9
Inflammatory Markers	Erythrocyte Sedimentation Rate (ESR)	32	mm/hr	0 – 30
	C-Reactive Protein (CRP)	1.20	mg/dL	0 – 3.00

Subsequent contrast-enhanced magnetic resonance imaging (MRI) of the abdomen with magnetic resonance cholangiopancreatography (MRCP) protocol revealed intrahepatic and extrahepatic biliary dilation with a distended CBD measuring 8 mm, compressed distally by an enlarged cystic duct containing a 9 mm filling defect suggestive of an obstructing gallstone (Figure 2). Representative MRI images are shown due to superior visualization of the pertinent findings. A low insertion of the cystic duct was again visualized. These findings were compatible with Type I Mirizzi syndrome. The pancreatic duct was normal in caliber.

**Figure 2:**
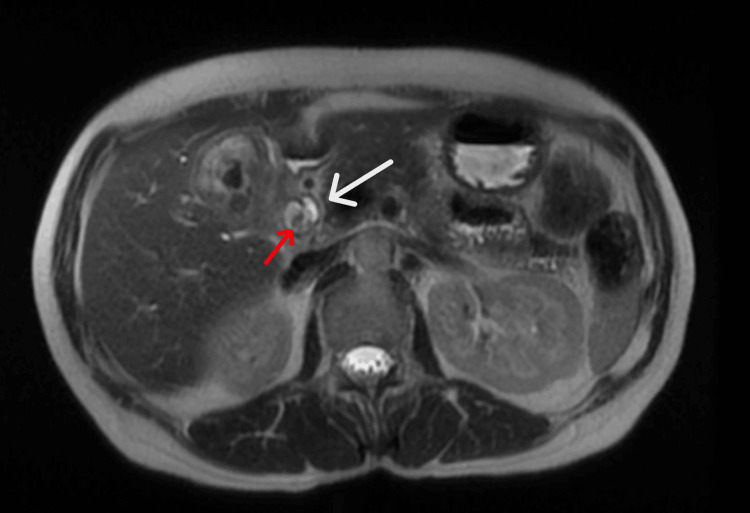
MRI of the Abdomen Demonstrating Features of Type I Mirizzi Syndrome MRI of the abdomen performed with and without intravenous contrast demonstrated intrahepatic and extrahepatic biliary ductal dilatation. The common bile duct measured 8 mm and exhibited distal extrinsic compression by an enlarged cystic duct containing a 9 mm filling defect (red arrow), consistent with an impacted gallstone. The cystic duct demonstrated low insertion anatomy, although the complete takeoff was not fully visualized. Overall findings were compatible with Type I Mirizzi syndrome (white arrow).

Gastroenterology was consulted, and the patient underwent ERCP with biliary sphincterotomy. Multiple stones were identified within the cystic duct and gallbladder. A plastic biliary stent was placed in the CBD to facilitate drainage.

Two days later, the patient underwent robotic-assisted cholecystectomy. Intraoperatively, severe inflammation and markedly distorted biliary anatomy were encountered, consistent with advanced chronic cholecystitis in the setting of Mirizzi syndrome. The stomach and duodenum were densely adherent to the gallbladder and required meticulous dissection to safely separate them from the operative field. The gallbladder was extremely contracted, densely fibrotic, partially intrahepatic, and filled with multiple gallstones. Indocyanine green (ICG) fluorescent cholangiography was utilized to aid in delineation of biliary structures and demonstrated the CBD; however, the cystic duct takeoff could not be safely identified because of severe inflammatory distortion and the patient’s low-inserting cystic duct anatomy. Although ICG cholangiography improved visualization of the CBD, it did not permit safe identification of the cystic duct takeoff, reinforcing the decision to proceed with subtotal fenestrating cholecystectomy. The gallbladder was fenestrated open, and multiple stones were removed. A dome-down subtotal fenestrating cholecystectomy was therefore performed to minimize the risk of bile duct injury. At the level of the infundibulum, the gallbladder remnant was intentionally left open, as no bile leakage was observed on either white light inspection or ICG cholangiography, and definitive identification and closure of the cystic duct orifice could not be safely achieved. The operative field was copiously irrigated to remove residual debris and evaluate for retained stones. A surgical drain was placed in the right upper quadrant at the conclusion of the procedure.

The postoperative course was uneventful, and the patient was discharged on an oral regimen of amoxicillin-clavulanate. Repeat CA 19-9 obtained following biliary decompression decreased from 114 U/mL to 27.8 U/mL (0.0-30.9 U/mL), supporting a benign obstructive etiology rather than underlying malignancy.

Seven weeks after cholecystectomy, the patient underwent elective removal of the plastic biliary stent. Within 48 hours of stent removal, she re-presented with acute onset right upper quadrant abdominal pain, described as sharp and constant, accompanied by nausea, multiple episodes of non-bilious vomiting, and decreased oral intake. She also reported low-grade fevers and generalized malaise. On examination, her temperature was 99.3°F, heart rate 115 beats per minute, respiratory rate 17 breaths per minute, blood pressure 119/77 mmHg, and oxygen saturation 97% on room air. The patient appeared uncomfortable but was alert and oriented. Abdominal examination revealed moderate right upper quadrant tenderness with voluntary guarding, without rebound tenderness or signs of peritonitis. No palpable masses or hepatosplenomegaly were appreciated. The remainder of the physical exam was unremarkable.

Laboratory evaluation revealed hyponatremia (sodium 128 mmol/L), total bilirubin elevation to 6.2 mg/dL, and a cholestatic pattern of liver injury with AST 221 U/L, ALT 388 U/L, and ALP 386 U/L. Leukocytosis was present with a white blood cell count of 17.8 × 10³/µL with marked neutrophilic predominance, consistent with an acute infectious or inflammatory process (Table 4). Lactic acid was elevated at 2.2 mmol/L. Broad-spectrum antibiotics with piperacillin-tazobactam were re-initiated.

**Table 4:** Comprehensive Metabolic Panel and Complete Blood Count with Differential

Test Category	Test	Value	Units	Reference Range
Complete Blood Count (CBC)	White Cell Count (WBC)	17.8	×10³/µL	4.0 – 10.5
	Hemoglobin	14.2	g/dL	11.2 – 15.7
	Platelets	156	×10³/µL	150 – 450
CBC with Differential	Immature Granulocytes	0.6	%	0.0 – 0.4
	Neutrophils	92.3	%	34.0 – 71.1
	Lymphocytes	2.6	%	19.3 – 51.7
	Monocytes	3.9	%	4.7 – 12.5
	Eosinophils	0.2	%	0.7 – 5.8
	Basophils	0.4	%	0.1 – 1.2
Comprehensive Metabolic Panel (CMP)	Sodium	128	mmol/L	136 – 145
	Potassium	3.6	mmol/L	3.5 – 5.1
	Chloride	94	mmol/L	98 – 107
	Carbon Dioxide	22.3	mmol/L	21.0 – 32.0
	Blood Urea Nitrogen (BUN)	13	mg/dL	7 – 18
	Creatinine	0.5	mg/dL	0.6 – 1.0
	Glucose	158	mg/dL	74 – 106
	Total Bilirubin	6.2	mg/dL	0.2 – 1.0
	Aspartate Aminotransferase (AST)	221	U/L	15 – 37
	Alanine Aminotransferase (ALT)	388	U/L	12 – 56
	Alkaline Phosphatase (ALP)	386	U/L	45 – 117
	Albumin	4.1	g/dL	3.4 – 5.0

Repeat contrast-enhanced CT of the abdomen and pelvis (Figure 3) demonstrated a complex fluid collection in the gallbladder fossa consistent with a biloma, along with biliary dilation; the CBD measured 12 mm. A small distal CBD filling defect and mural thickening were also noted (Figure 4). Right upper quadrant ultrasound (Figure 5) confirmed a 5.1 × 3.2 × 3.2 cm complex fluid collection most consistent with a biloma in the appropriate clinical context. CBD dilation (11 mm) was again noted.

**Figure 3:**
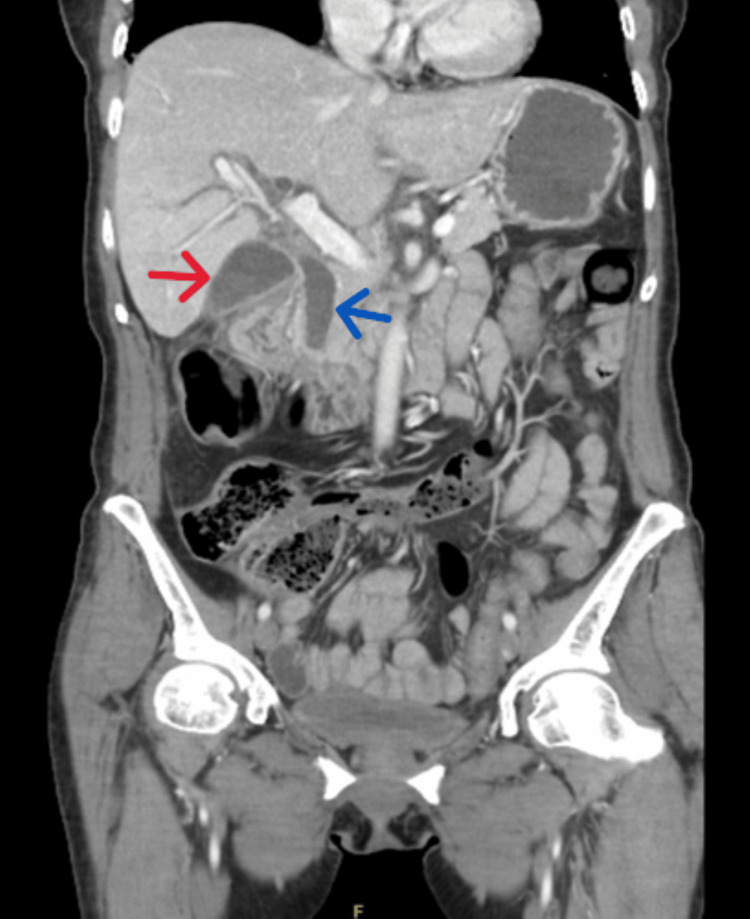
Contrast-enhanced computed tomography (CT) scan of the abdomen and pelvis Contrast-enhanced CT of the abdomen and pelvis demonstrating a complex fluid collection in the gallbladder fossa, consistent with a biloma (red arrow). Associated biliary dilatation is present, involving both intrahepatic and extrahepatic bile ducts. The common bile duct measures 12 mm in diameter (blue arrow).

**Figure 4:**
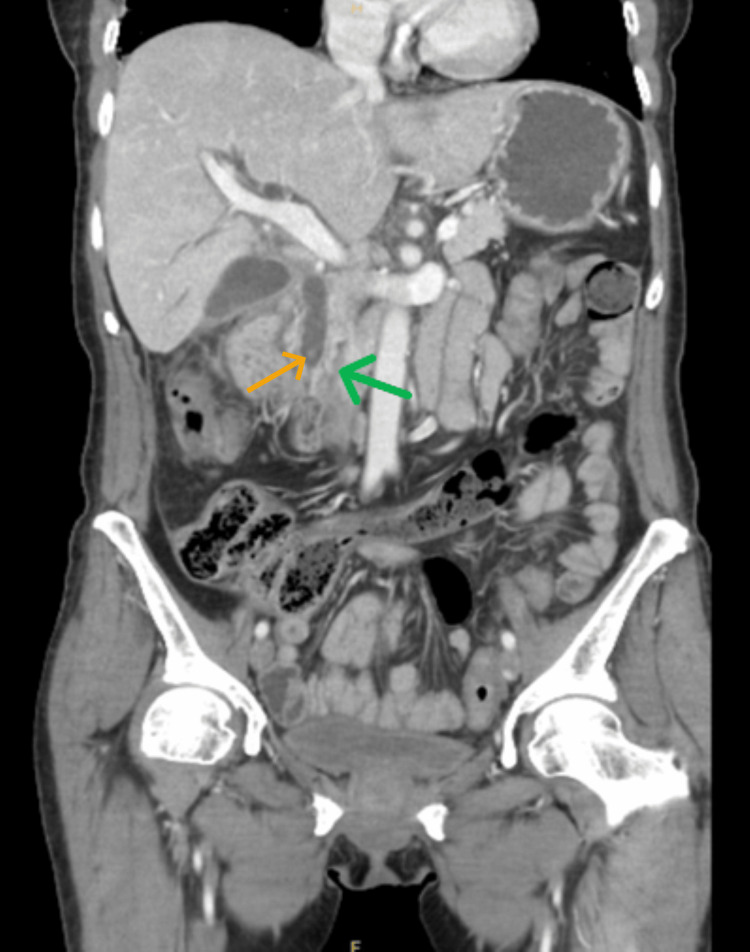
Contrast-enhanced computed tomography (CT) scan of the abdomen and pelvis Contrast-enhanced CT of the abdomen and pelvis demonstrating a small (2 mm) filling defect (orange arrow) within the distal common bile duct with associated mural thickening (green arrow). These findings were concerning for a retained distal CBD stone (orange arrow) with associated inflammatory mural thickening (green arrow).

**Figure 5:**
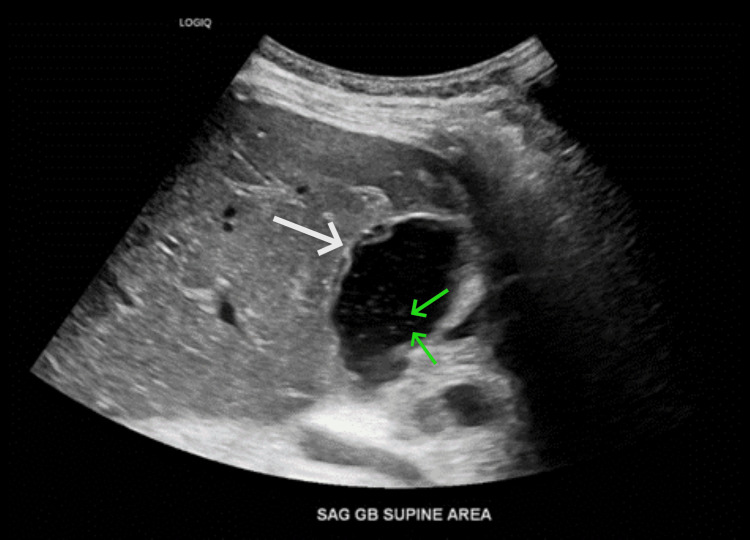
Right upper quadrant ultrasound (US) Right upper quadrant ultrasound demonstrating a 5.1 × 3.2 × 3.2 cm complex fluid collection (white arrow) within the gallbladder fossa containing internal echogenic debris/sludge (green arrows), concerning for biloma in the appropriate clinical setting.

Interventional Radiology performed ultrasound- and fluoroscopy-guided percutaneous drainage of the biloma (Figures 6, 7), yielding approximately 200 mL of bilious fluid, supporting the diagnosis of bile leakage. No discrete site of active bile extravasation was definitively demonstrated on follow-up imaging or subsequent ERCP. A pigtail catheter was placed for continuous drainage (Figure 7). Given persistent biliary dilation and concern for obstruction, Gastroenterology was re-consulted. The patient underwent repeat ERCP with endoscopic ultrasound (EUS), which identified a retained CBD stone causing recurrent biliary obstruction; the stone was successfully extracted. Following endoscopic intervention, biloma output decreased significantly. Repeat contrast-enhanced CT confirmed decompression of both the biloma and biliary tree.

**Figure 6:**
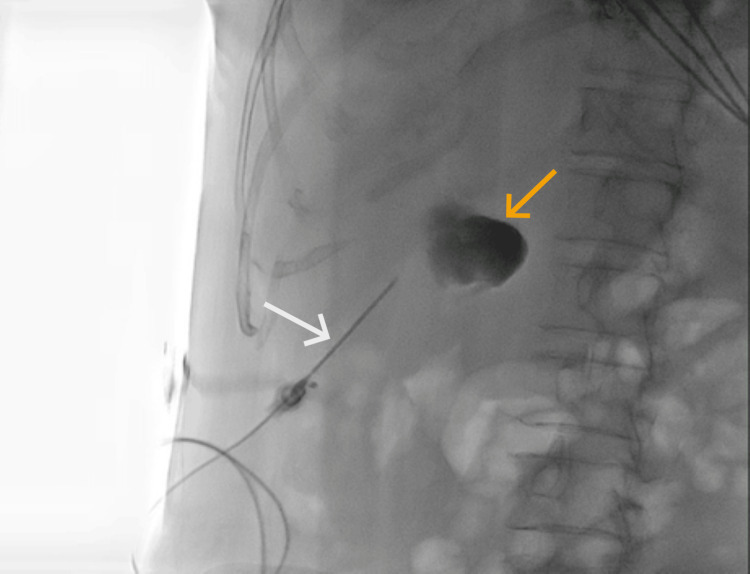
Fluoroscopy-Guided Needle Access of Gallbladder Fossa Biloma Fluoroscopy-guided access of the gallbladder fossa fluid collection demonstrating percutaneous needle placement (white arrow) into the biloma cavity under imaging guidance. Contrast injection outlines the collection (orange arrow) without definite communication with the biliary tree.

**Figure 7:**
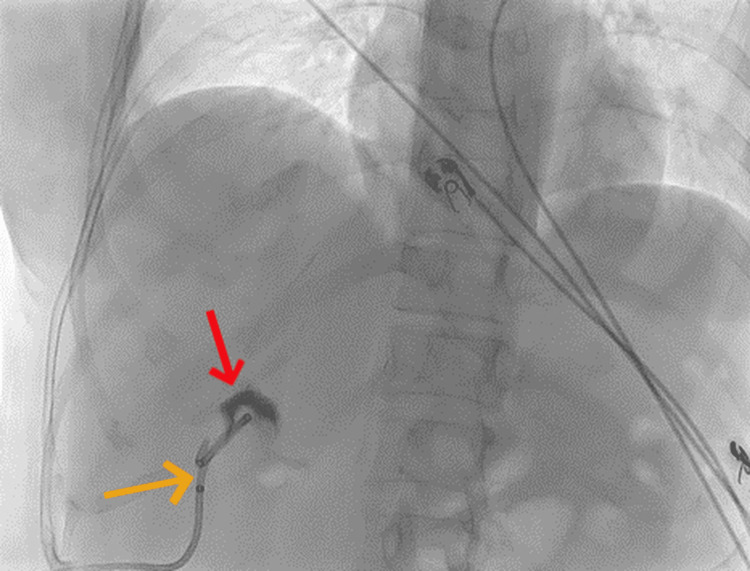
Fluoroscopy-Guided Percutaneous Drainage of Gallbladder Fossa Biloma Fluoroscopy-guided percutaneous drainage of the gallbladder fossa biloma demonstrating placement of an 8 French pigtail catheter (orange arrow) within the fluid collection (red arrow). Approximately 200 mL of green bilious fluid was aspirated, supporting postoperative bile leakage. No definite communication with the biliary tree was demonstrated during the procedure.

Cultures of the drained bilious fluid grew *Klebsiella pneumoniae*, and blood cultures yielded *Escherichia coli*, consistent with biliary sepsis. The patient improved clinically with intravenous antibiotics and source control through biloma drainage and biliary decompression and was subsequently discharged on oral cefdinir based on antimicrobial susceptibility results and continued clinical stability. Outpatient follow-up was arranged with General Surgery for drain removal.

## Discussion

Mirizzi syndrome is a rare complication of chronic cholelithiasis characterized by extrinsic compression of the CHD or CBD by an impacted gallstone. The Csendes classification system stratifies disease severity according to the degree of biliary fistula formation and helps guide operative management [1].

Clinically, Mirizzi syndrome presents a diagnostic dilemma due to its overlapping features with biliary tract malignancies, especially cholangiocarcinoma [2-4]. Imaging findings, such as irregular gallbladder wall thickening, biliary dilation, or ductal obstruction, may be indistinguishable from neoplastic processes. This overlap is further complicated when tumor markers, such as CA 19-9, are elevated. While CA 19-9 is classically associated with pancreaticobiliary malignancies, benign conditions like obstructive jaundice or cholangitis may also produce elevated levels [6-8]. In our patient, the presence of weight loss, abnormal gallbladder wall thickening on CT, and elevated CA 19-9 initially raised concern for malignancy, prompting a comprehensive oncologic evaluation.

Several findings initially favored a malignant process, including unintentional weight loss, irregular gallbladder wall thickening with enhancement on CT imaging, biliary ductal dilation, and elevated CA 19-9. However, subsequent MRI with MRCP protocol demonstrated extrinsic compression of the biliary tree by an enlarged cystic duct containing a filling defect consistent with an obstructing gallstone, findings that strongly supported Type I Mirizzi syndrome rather than cholangiocarcinoma. The absence of a discrete biliary mass lesion and the subsequent normalization of CA 19-9 following biliary decompression further supported a benign obstructive etiology. Elevated CA 19-9 levels occur commonly in benign biliary obstruction, which limits its specificity in differentiating malignant from benign disease [9]. Accordingly, CA 19-9 should be interpreted with caution in the setting of biliary obstruction. In our patient, repeat CA 19-9 following biliary decompression decreased from 114 U/mL to 27.8 U/mL, further supporting a benign obstructive etiology rather than malignancy.

In the diagnostic algorithm, imaging plays a central role. As highlighted by Seah et al., abdominal US and CT have limited sensitivities for detecting Mirizzi syndrome, approximately 11.4% and 40%, respectively [4]. MRCP demonstrates higher sensitivity (88.9%) and provides superior anatomic resolution of the biliary tree [4], although it lacks therapeutic capability. These findings are generally consistent with prior published series demonstrating superior diagnostic performance of MRCP compared with ultrasound and CT in the evaluation of Mirizzi syndrome [2,4]. In our case, MRI with MRCP protocol was instrumental in identifying cystic duct obstruction and extrinsic compression of the biliary tree, confirming the diagnosis of Type I Mirizzi syndrome. However, despite its invasiveness and procedural risks, ERCP remains the most definitive diagnostic and therapeutic modality, with a diagnostic accuracy approaching 92.5% [2,10]. ERCP not only confirms the diagnosis but also allows for therapeutic interventions such as stone extraction and biliary stenting. Additionally, intraoperative ICG fluorescent cholangiography, as utilized in our case, is an emerging adjunct to better delineate biliary anatomy and reduce bile duct injury during complex cholecystectomies [11].

Surgical intervention remains the definitive treatment for Mirizzi syndrome. Significant inflammation and distorted anatomy in Mirizzi syndrome elevate the risk of bile duct injury, supporting the use of subtotal fenestrating cholecystectomy, as demonstrated in our patient. Minimally invasive approaches, including laparoscopic and robotic techniques, have increasingly been utilized in selected patients when expertise and appropriate resources are available, potentially providing reduced morbidity compared to open surgery [1]. In this case, a robotic-assisted subtotal fenestrating cholecystectomy with an intentionally open remnant was performed because the cystic duct takeoff could not be safely identified despite ICG cholangiography, highlighting the importance of adapting surgical strategy to intraoperative findings.

In general, in Type I disease, open or laparoscopic cholecystectomy is typically sufficient [12]. For Types II through IV, the presence of cholecystocholedochal fistulas or severe ductal involvement may necessitate more complex biliary reconstruction, including partial cholecystectomy with ductal repair, choledochoplasty, or Roux-en-Y hepaticojejunostomy [1,13,14]. Surgical management strategies according to the Csendes classification system are summarized in Table 5.

**Table 5:** Csendes classification system for Mirizzi Syndrome

Type	Description	Surgical Approach	References
I	External compression of the common hepatic duct (CHD) by a gallstone impacted in the cystic duct or gallbladder neck, without fistula formation	Cholecystectomy (laparoscopic or robotic approaches may be attempted in selected cases; high conversion rates may occur in severe inflammation) ± choledochostomy with T-tube placement	[15-17]
II	Cholecysto-biliary fistula involving less than one-third of the circumference of the CHD	Partial cholecystectomy with primary suture repair of the fistula using absorbable material, or choledochoplasty using the gallbladder remnant wall ± T-tube placement distal to the fistula	[15,18]
III	Cholecysto-biliary fistula involving up to two-thirds of the circumference of the CHD	Partial cholecystectomy with choledochoplasty (primary suture repair generally not recommended because of the extent of duct involvement) ± T-tube placement	[15,18]
IV	Complete destruction of the CHD wall due to a cholecysto-biliary fistula	Bilioenteric reconstruction with Roux-en-Y hepaticojejunostomy	[15,18]
Va	Cholecystoenteric fistula without gallstone ileus in conjunction with any of the types above	Cholecystectomy with fistula takedown and enteric repair, in addition to management of the biliary component according to the corresponding Mirizzi subtype	[19,20]
Vb	Cholecystoenteric fistula with gallstone ileus in conjunction with any of the types above	Enterolithotomy (stone extraction) ± fistula takedown and cholecystectomy (single-stage or staged approach), with biliary reconstruction according to the corresponding Mirizzi subtype	[19,20]

This case highlights several important clinical considerations. First, the patient’s presentation with weight loss, elevated CA 19-9, and imaging findings concerning for malignancy underscores the diagnostic challenge of distinguishing Mirizzi syndrome from cholangiocarcinoma. Second, it demonstrates the importance of a staged, multidisciplinary approach incorporating ERCP, surgical intervention, and careful postoperative management. Finally, this case highlights the potential for complex postoperative biliary complications requiring continued vigilance even after apparent clinical resolution.

Postoperative complications, while uncommon, can be clinically significant. In this case, the patient developed a biloma, a localized collection of bile outside the biliary tree. Bilomas can occur following invasive hepatobiliary procedures, including ERCP and cholecystectomy, and are typically the result of iatrogenic bile leaks. The etiology in this case was likely multifactorial, with recurrent biliary obstruction and increased intraductal pressure from a retained CBD stone likely serving as major contributors in the setting of prior complex biliary surgery and recent biliary stent removal. Although symptom onset occurred shortly after stent removal, a direct causal relationship could not be definitively established. 

Additionally, subtotal fenestrating cholecystectomy itself may predispose to postoperative bile leakage or retained stones because of the intentionally incomplete nature of the resection and inability to achieve definitive cystic duct closure in severely inflamed anatomy. In our patient, the cystic duct takeoff could not be safely identified intraoperatively despite ICG cholangiography, necessitating an open fenestrated remnant and placement of a surgical drain. These operative factors, together with subsequent retained CBD stone and recurrent biliary obstruction, likely contributed to the patient’s later infected biloma formation. Although a discrete site of active bile extravasation was not definitively identified on repeat ERCP or follow-up imaging, aspiration of bilious fluid from the gallbladder fossa strongly supported the presence of postoperative bile leakage. If not promptly recognized, bilomas may become secondarily infected and contribute to systemic illness. In our patient, the subsequent identification of a retained CBD stone highlighted the importance of considering ongoing biliary obstruction as a contributing factor.

The retained CBD stone likely played a central role in the patient’s recurrent biliary dilation, cholestatic liver injury, infected biloma formation, and subsequent biliary sepsis by contributing to recurrent biliary obstruction and increased intraductal pressure. The precise origin of the retained stone remains uncertain. Potential mechanisms include incomplete extraction during the initial ERCP, interval migration from the gallbladder or cystic duct remnant following subtotal fenestrating cholecystectomy, or, less likely, de novo stone formation during the postoperative period. Given the severe inflammatory distortion encountered intraoperatively and inability to definitively identify the cystic duct anatomy, interval migration from residual cystic duct or gallbladder remnant stones may have been particularly contributory in this case.

Additionally, the patient’s clinical course was complicated by biliary sepsis with *Klebsiella pneumoniae* isolated from biloma fluid cultures and *Escherichia coli* identified in blood cultures.

This report has several limitations inherent to single-case studies. Although the temporal relationship between biliary stent removal and subsequent infected biloma formation was notable, direct causality could not be definitively established. Additionally, no discrete site of active bile extravasation was identified on repeat ERCP or follow-up imaging. Nevertheless, the combination of operative findings, bilious drainage, retained CBD stone, and subsequent clinical course provides important insight into the multifactorial mechanisms that may contribute to postoperative biliary complications in complex Mirizzi syndrome.

Management of iatrogenic bilomas is typically multimodal. Initial treatment involves percutaneous drainage, usually under ultrasound and fluoroscopic guidance, coupled with broad-spectrum antibiotic therapy [5,21]. In cases with ongoing obstruction or persistent bile leak, ERCP with biliary sphincterotomy and/or stent placement may be required [22,23]. Surgical re-exploration is reserved for refractory or high-grade injuries [21]. If untreated, bilomas can progress to abscess formation, biliary peritonitis, or sepsis, emphasizing the importance of timely diagnosis and intervention. Minimally invasive approaches have improved management outcomes, but treatment should be individualized based on the size of the biloma, presence of infection, and patient comorbidities.

Written informed consent was obtained from the patient for publication of this case report and accompanying images.

## Conclusions

Mirizzi syndrome is an uncommon but clinically significant biliary pathology that requires a high index of suspicion because of its ability to mimic hepatobiliary malignancies. This case highlights the diagnostic complexity of Mirizzi syndrome, particularly in the presence of concerning clinical features, abnormal imaging findings, and elevated tumor markers that may initially suggest malignancy. Accurate diagnosis hinges on a structured, multidisciplinary approach integrating clinical findings with advanced imaging and endoscopic evaluation. This case also underscores the importance of recognizing infected biloma formation as a potential complication following complex biliary interventions, particularly in patients undergoing subtotal fenestrating cholecystectomy and biliary stent management. In our patient, a retained CBD stone likely contributed to recurrent biliary obstruction, cholestatic liver injury, infected biloma formation, and biliary sepsis. Clinicians should maintain a high index of suspicion for retained stones, recurrent biliary obstruction, bile leak, and biloma in patients who develop cholestasis, abdominal pain, or sepsis following subtotal cholecystectomy, biliary stent removal, or other complex biliary interventions. Early recognition, prompt drainage, and timely endoscopic management are essential to prevent infectious sequelae and optimize clinical outcomes. In patients undergoing subtotal cholecystectomy and biliary stent management, particularly in the setting of complex biliary anatomy or retained stones, clinicians should maintain close clinical follow-up and a low threshold for repeat imaging or endoscopic evaluation when new abdominal pain, cholestasis, or signs of sepsis develop.
